# Exacerbation of *Mycobacterium avium* pulmonary infection by comorbid allergic asthma is associated with diminished mycobacterium-specific Th17 responses

**DOI:** 10.1080/21505594.2021.1979812

**Published:** 2021-10-04

**Authors:** Yeeun Bak, Sang Chul Park, Dahee Shim, Yura Ha, Jumi Lee, Hongmin Kim, Kee Woong Kwon, Joo-Heon Yoon, Sung Jae Shin

**Affiliations:** a Department of Microbiology, Yonsei University College of Medicine, Seoul, Korea; b Graduate School of Medical Science, Brain Korea 21 Project, Yonsei University College of Medicine, Seoul, Korea; c Department of Otorhinolaryngology-Head and Neck Surgery, Kangnam Sacred Heart Hospital, Hallym University College of Medicine, Seoul, Korea; d Department of Otorhinolaryngology, Yonsei University College of Medicine, Seoul, Korea; e Global Research Laboratory for Allergic Airway Diseases, Yonsei University College of Medicine, Seoul, Korea; f The Airway Mucus Institute, Yonsei University College of Medicine, Seoul, Korea; g Department of Microbiology, Institute for Immunology and Immunological Diseases, Yonsei University College of Medicine, Seoul, Korea

**Keywords:** Nontuberculous mycobacteria, *Mycobacterium avium*, allergic asthma, Th17 response, chronic respiratory disease, comorbid diseases, pathogenesis

## Abstract

Accumulating evidence suggests that two chronic respiratory diseases, nontuberculous mycobacterium (NTM)-pulmonary disease (PD) and allergic asthma, are frequently present together and that they likely influence the disease development and progression of each other. However, their precise interactions regarding the pathogenesis of comorbid diseases versus that of individual diseases are not well understood. In this study, comorbid diseases (*i.e., Mycobacteria avium* (Mav) pulmonary infection (PI) (Mav-PI) and ovalbumin-induced allergic asthma) were established in mice in different orders and at different time periods. Individual disease-specific characteristics, including alterations in immune cell populations and antigen-specific immune responses, were analyzed and compared. To assess Mav-PI pathogenesis, lung inflammation and bacterial burden levels were also determined. Allergic asthma induction in the presence of Mav-PI markedly aggravated Mav-PI pathogenesis by increasing the bacterial burden and the severity of lung inflammation. Interestingly, the general outcome of allergic asthma with goblet cell hyperplasia was alleviated at a chronic stage in the comorbid mouse model. Overall, the increase in the number of Mav CFUs was inversely correlated with the Mav-specific Th17 response, as confirmed by comparing BALB/c and C57BL/6J mice. Overall, the pathogenesis of existing Mav-PI is more severely affected by allergen exposure than vice versa. This Mav-PI exacerbation is associated with disruption of Mav-specific Th17 responses. This study provides the first evidence that the Mav-specific Th17 response plays an important role in the control of Mav pathogenesis in the presence of allergic asthma, indicating that targeting the Th17 response has therapeutic potential for NTM-PD accompanied by allergic asthma.

## INTRODUCTION

Nontuberculous mycobacteria (NTMs) are a collection of mycobacterial species other than the *Mycobacterium tuberculosis* (Mtb) complex and *Mycobacterium leprae*. NTMs have emerged as representative opportunistic pathogens that are ubiquitous [1,2]. The global prevalence of NTM infection has recently increased, and the number of patients with NTM-associated pulmonary diseases (NTM-PDs) has gradually increased [3,4]. The *Mycobacterium avium* (Mav) complex is the most prevalent pathogen among NTMs worldwide and causes NTM-PD [5,6].

Although NTMs have received considerable attention from researchers, the available preventative and treatment regimens for NTM-associated pulmonary infection (PI) (NTM-PI) remain unsatisfactory [7–9]. The most notable problem is that the progression of NTM-related infectious disease is enhanced in vulnerable and immunocompromised patients and in those with underlying comorbidities [10,11]. For example, patients with acquired immune deficiency syndrome and, in particular, those who suffer from other respiratory diseases exhibit increased susceptibility to NTM infections [12]. Among these diseases, chronic respiratory diseases, including allergic asthma, are regarded as critical risk factors for NTM-PD [13,14]. Patients with asthma and using inhaled corticosteroids (ICSs) might be at significantly elevated risk for NTM-PD [15]; conversely, patients with allergic asthma who are infected with NTMs show a severe asthma phenotype, which hinders control of airway obstruction despite treatment [16,17].

Asthma is a chronic airway disease characterized by variable airway obstruction associated with airway hyperresponsiveness [18] and typically represents a type 2 immune response [19]. ICSs are the immunosuppressants most widely used to control allergic asthma. However, ICSs predispose patients to infection by disrupting their defense systems. NTM-PD is frequently accompanied by chronic airway disease, such as allergic asthma; notably, both have similar symptoms and are common in elderly women [20–22]. The coexistence of NTM-PI and allergic asthma is likely to accelerate the progression of both diseases; thus, a general understanding of comorbid conditions regarding pathology and immunology is clinically important. Although many studies have revealed mycobacterial infection-related conditions comorbid with allergic asthma from various perspectives of allergic asthma [23,24], only a few studies have investigated immunological progression [25,26]. Moreover, to our knowledge, whether these chronic diseases influence one another has not been investigated in previous experimental studies or clinical settings.

To address the abovementioned issues, we investigated PD due to the comorbidity of NTM-PI and allergic asthma. This study aimed to analyze whether the bacterial growth rate, pathological outcomes and immunologic profiles are altered by the underlying diseases and to examine the pathogenesis of NTM-PI comorbid with allergic asthma.

## MATERIALS AND METHODS

### Animals and Ethics Statement

Specific pathogen-free female BALB/c and C57BL/6 J mice (aged 5 to 7 weeks) were purchased from Japan SLC, Inc. (Shinzuoka) and maintained under specific pathogen-free conditions. The use of animals in this study was approved by the Institutional Animal Care and Use Committee (permit number: 2017–0342, 2020–0011) of the Yonsei University College of Medicine, and the study was conducted according to the guidelines for animal experiments established by the International Association of Veterinary Editors.

### Experimental Animal Models

To investigate how an individual disease influences another disease depending on the induction order and timing, the mice were divided into four groups: the naïve, allergic asthma, Mav-infected, and comorbid groups. To develop comorbid groups in different orders, Mav was infected before allergic asthma (Mav infection first, MavF) or after allergic asthma (allergic asthma first, AF) induction. The details of the mouse models are described in the Supplementary Methods.

### Mav Culture and Culture Filtrate Antigen (Mav_Ag_) Preparation

The Mav subspecies *hominissuis* clinical strain SM#7 (hereafter referred to as Mav), which was isolated from a patient with typical NTM-PD at Samsung Medical Center (Seoul, Korea), was used. Mav was cultivated in Middlebrook 7H9 broth (BD-Difco, Le Pont de Claix, France) supplemented with oleic acid-albumin-dextrose-catalase (OADC) at 37°C, and the colony-forming units (CFUs) were quantified on Middlebrook 7H10-OADC agar [27]. Mav_Ag_ was obtained by concentrating the culture supernatant after Mav was grown in protein-free modified Watson-Reid (mWR, pH 6.0) medium to the mid-logarithmic phase as previously described (Supplementary Methods) [28,29].

### Bacterial Infection

After bacteria were precultured for one week in 7H9-OADC broth, the collected pellet was washed with PBS to remove residual medium. Bacteria resuspended in 4 mL of PBS were used to infect mice via aerosolization using an inhalation exposure system (Glas-Col, Terre Haute, IN, United States). To confirm the absolute infection rate, randomly selected infected mice were sacrificed four hours later. The right lung lobe was homogenized, and the homogenate was serially diluted and plated onto 7H10-OADC agar plates. After two weeks, CFUs were quantified to determine the approximate initial infection dose (initial infection dose of all experiments in this article = 10^4.5^ ~ 10^4.7^ CFUs) [30].

### Bacterial Counts and Assessment of Lung Inflammation

Serially diluted lung homogenates were plated on Middlebrook 7H10-OADC agar for quantification of viable bacteria. Paraffin-embedded lung tissue sections were stained with hematoxylin and eosin (H&E) or periodic acid-Schiff (PAS) solution. Lung inflammation was graded using ImageJ software (version 1.48k, NIH), and goblet cell hyperplasia was graded using a previously reported semiquantitative scoring system. In brief, photos of H&E-stained lung sections were converted into grayscale, and the inflamed areas were marked as black in contrast to noninflamed areas marked as white. The percentage of the inflamed lung area was determined by using ImageJ. To quantify airway goblet cells, the following five-point grading system was used: 0, <5% PAS-positive cells; 1, 5–25%; 2, 25–50%; 3, 50–75%; and 4, >75%. Analyses were performed in a blinded manner, and slides were presented in random order for each examination [31,32].

### Flow Cytometry and Cytokine Quantification

For analysis of cell types, suspended single cells were stained as previously described [33]. After the surface molecules were stained, the cells were fixed with IC Fixation Buffer (Invitrogen) [34]. The stained cells were analyzed by flow cytometry using FlowJo software (TreeStar, Inc.). For assessment of cytokine levels, supernatants from specific antigen-restimulated cells were analyzed with enzyme-linked immunosorbent assays (ELISAs). The antibody details are provided in Supplementary Tables 1 and 2, and representative flow cytometry plots are shown in Supplementary Figure 1. The details for the intracellular staining of antigen-specific T cell subsets are provided in the Supplementary Methods.

### Serum Antibody Titration

Serum antigen-specific antibodies were quantified by ELISA as previously described [31]. Briefly, separated serum was diluted (1:50 for ovalbumin (OVA)-specific IgE and 1:10,000 for OVA-specific IgG1) and added to a plate coated with OVA (2 μg/mL). The antibody titers were then detected with biotin-conjugated rat anti-mouse IgE or biotin-conjugated rat anti-mouse IgG1 (BD).

### Statistical Analysis

All data represent at least two or three independent experiments, and four-to-seven mice were included in each group in each experiment. Between-group differences were analyzed via unpaired *t*-tests. The results among more than two groups were compared by one-way analysis of variance followed by Tukey’s multiple comparison test. The correlation between CFU levels and cytokine-specific T cell frequencies was analyzed by linear regression. All statistical analyses were conducted using Prism (GraphPad Software version 7.0). Statistical significance was determined and is indicated as follows: *p < 0.05, **p < 0.01, and ***p < 0.001.

## RESULTS

### Aggravation of Pathogenesis due to Mav-Related PI (Mav-PI) Followed by Allergic Asthma

**Figure 1.**
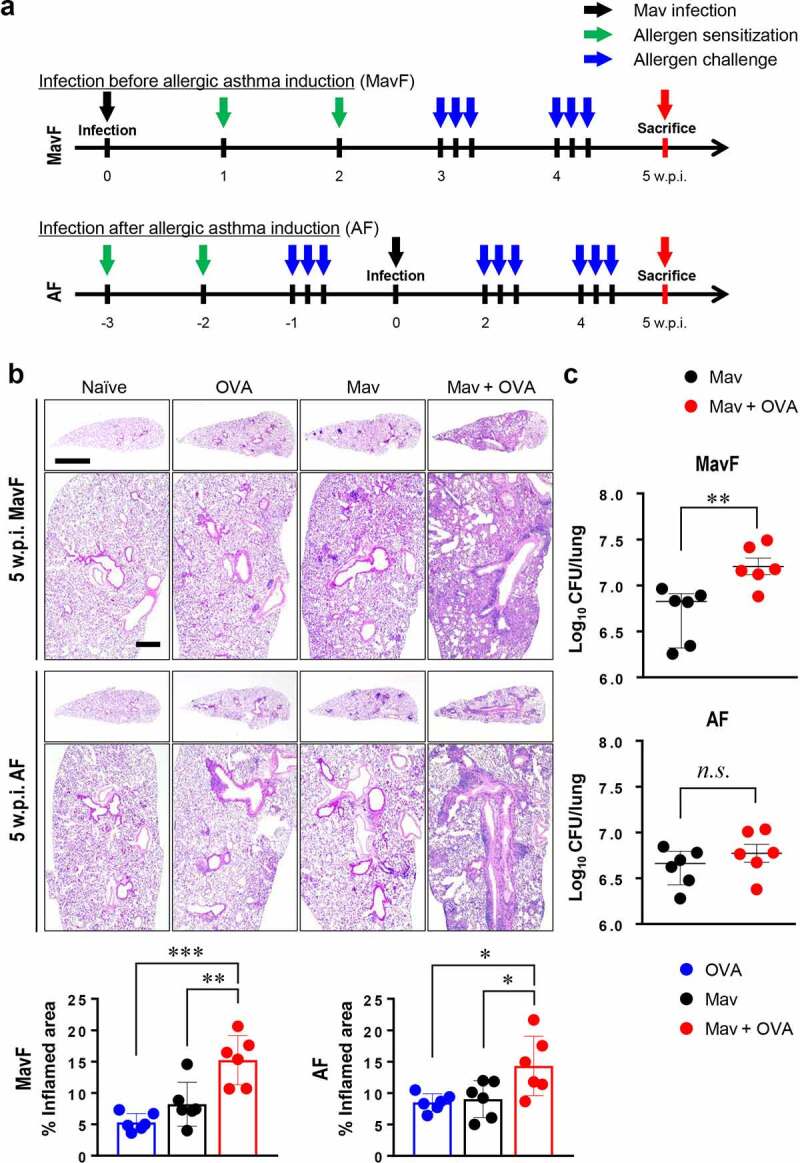
**Progression of *Mycobacterium avium* (Mav) infection with allergic asthma in BALB/c mice at 5 weeks post infection (w.p.i.)**. (a) Experimental protocol for the development of a comorbid condition via Mav infection before allergic asthma induction (Mav infection first, MavF) or after allergic asthma induction (allergic asthma first, AF). The mice were intraperitoneally sensitized with ovalbumin (OVA) (green arrow) and challenged by aerosol spray (blue arrow) before or after aerogenic Mav infection (black arrow). Each group was sacrificed at 5 w.p.i. (red arrow). (b) Representative hematoxylin and eosin (H&E)-stained lung sections (original magnification: ×1, scale bar: 2 mm; ×4, scale bar: 500 µm) are shown. The dot plots present the quantified inflamed lung areas in the allergic asthma (blue circle), Mav-infected (black circle) and comorbid (red circle) groups. (c) The bacterial burden was compared between the Mav-infected and comorbid groups. Between-group differences were statistically compared by unpaired *t*-tests, and results among more than two groups were compared by one-way analysis of variance followed by Tukey’s multiple comparison test (*p< 0.05, **p< 0.01, ***p< 0.001, *n.s*., not significant)

To reflect clinical conditions in which NTM-PI and allergic asthma coexist, Mav-PI and allergic asthma were induced in different orders (Figure 1a and Supplementary Methods). At 5 weeks post infection (w.p.i.), the lungs of the infected and comorbid groups exhibited severe inflammatory lesions, unlike those of the naïve group (Figure 1b). Interestingly, inflammation was worse in the comorbid group than in the single-disease groups, regardless of the order in which the diseases were induced. In contrast to the pathological analysis results, bacterial burden analysis showed that the growth of Mav in the comorbid group was faster than that in the infected group in the MavF scheme but comparable to that in the infected group in the AF scheme (Figure 1c).

**Figure 2.**
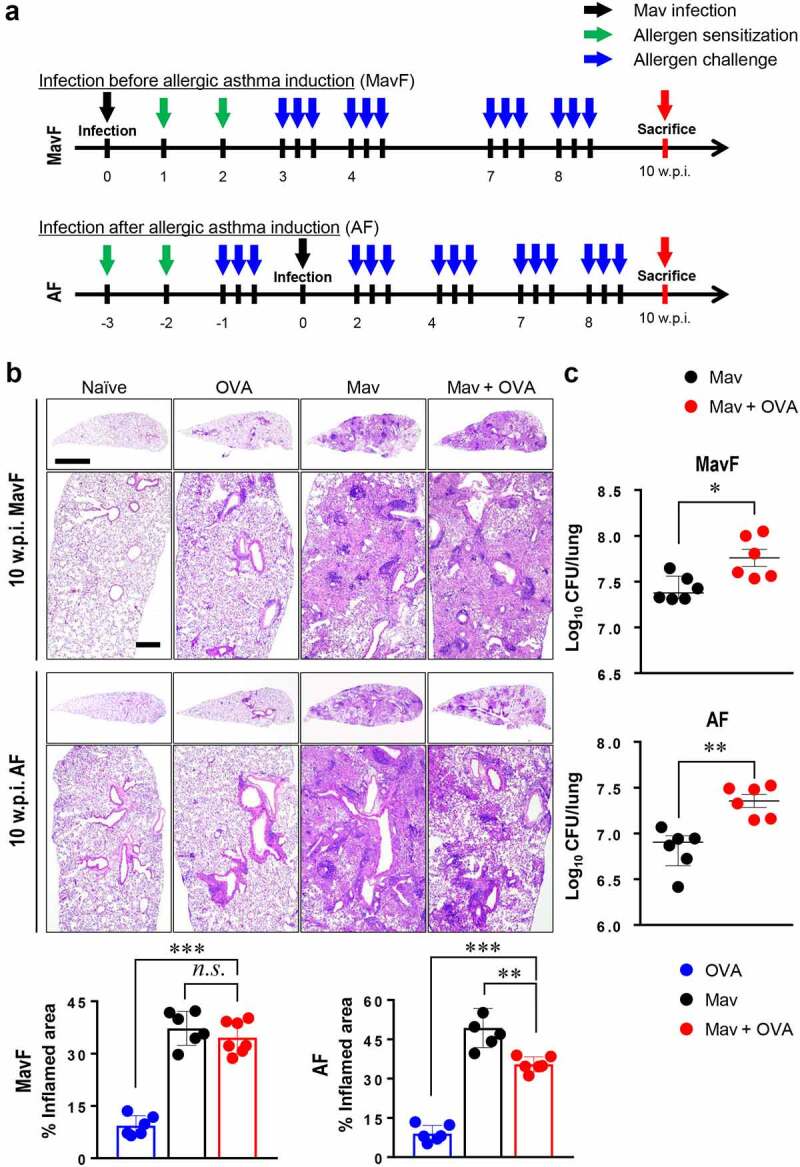
**Progression of *Mycobacterium avium* (Mav) infection with allergic asthma in BALB/c mice at 10 weeks post infection (w.p.i.)**. (a) The experimental protocol up to 10 w.p.i. for Mav infection is indicated in the disease timeline. The condition of each group of mice was established at 5 w.p.i., and the mice were sacrificed at 10 w.p.i. Aerosol challenges (blue arrow) were repeated after ovalbumin sensitization by intraperitoneal injection (green arrow) until all the mice were sacrificed. Ten weeks after Mav infection (black arrow), all the mice were sacrificed simultaneously (red arrow). (b) Representative hematoxylin and eosin (H&E)-stained lung sections (original magnification: ×1, scale bar: 2 mm; ×4, scale bar: 500 µm) are shown. The dot plots present the inflamed lung areas in the allergic asthma (blue circle), Mav-infected (black circle) and comorbid (red circle) groups, which were quantified using ImageJ software. (c) The bacterial burden was compared between the Mav-infected group (black circle) and the comorbid group (red circle). Between-group differences were statistically compared by unpaired *t*-tests, and results among more than two groups were compared by one-way analysis of variance followed by Tukey’s multiple comparison test (*p< 0.05, **p< 0.01, ***p< 0.001, *n.s*., not significant)

Mice were repeatedly challenged with OVA up to 10 w.p.i. to investigate whether the increased number of CFUs and aggravated lung inflammation during comorbid conditions at 5 w.p.i were alleviated (Figure 2a).

Lung inflammation and bacterial growth were much greater at 10 w.p.i. than at 5 w.p.i. Interestingly, at later time points, OVA challenge did not aggravate tissue damage despite increasing Mav proliferation at 10 w.p.i. The number of CFUs in the comorbid group in the AF scheme was increased at 10 w.p.i. in accordance with the degree of allergen induction (Figure 2b and 2c).

### Immune Cell Infiltration Was Comparable in the Comorbid and Mav-PI-Only Groups

**Figure 3.**
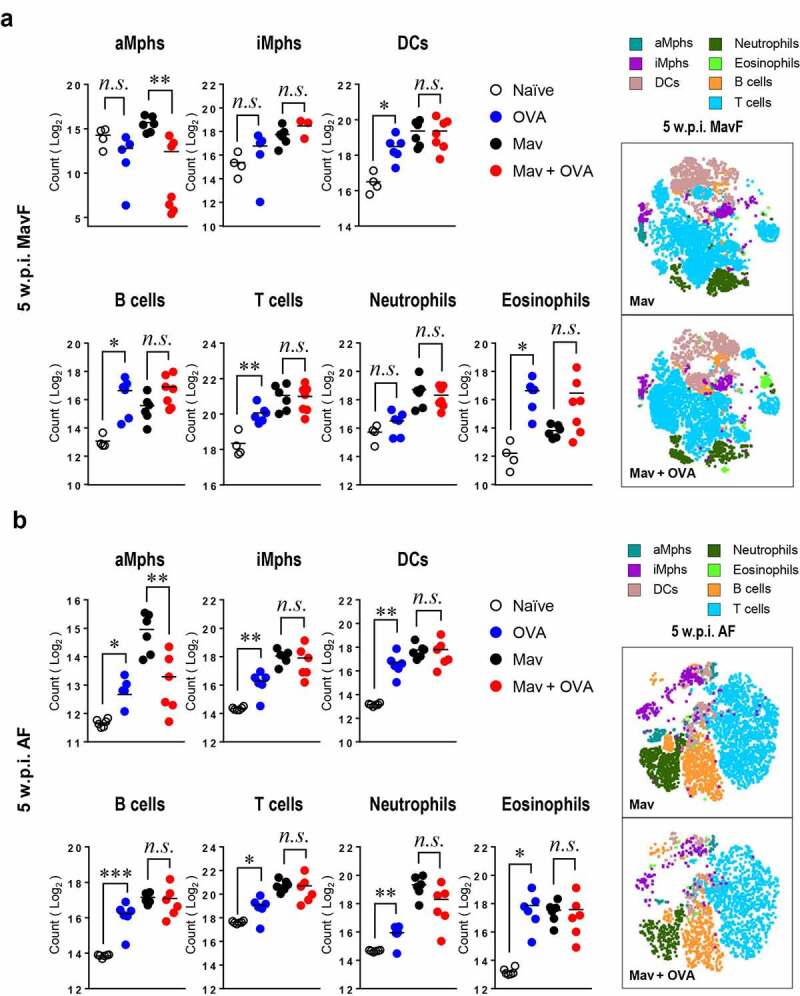
**Alteration of the cellular composition in the lungs by *Mycobacterium avium* (Mav) infection accompanied by allergic asthma in BALB/c mice at 5 weeks post infection (w.p.i.)**. All mice from each condition were simultaneously euthanized at 5 w.p.i. The numbers of immune cells, including alveolar macrophages (aMphs), interstitial macrophages (iMphs), dendritic cells (DCs), B cells, T cells, neutrophils and eosinophils, were determined among separated single cells from each group by flow cytometry. Representative immune cell populations collected at 5 w.p.i. before allergic asthma induction (Mav infection first, MavF) (a) and after allergic asthma induction (allergic asthma first, AF) (b) were analyzed. To verify the difference between the infected and comorbid groups, the t-stochastic neighbor embedding (t-SNE) results for each cell population were merged between the infected and comorbid groups. Between-group differences were statistically compared by unpaired *t*-tests, and results among more than two groups were compared by one-way analysis of variance followed by Tukey’s multiple comparison test (*p< 0.05, **p< 0.01, ***p< 0.001, *n.s*., not significant)

Based on the aggravated inflammation in the comorbid group, we hypothesized that marked immune cell infiltration in the lungs was responsible for the severe inflammation. However, unexpectedly, the numbers of immune cells were similar in the infected and comorbid groups at 5 w.p.i. (Figure 3) and even tended to be lower in the comorbid group at 10 w.p.i. (Supplementary Figure. 1a and 2).

In detail, there were fewer alveolar macrophages (aMphs), which are important for mycobacterial survival [35], in the comorbid group than in the infected group in both the MavF and AF schemes at 5 w.p.i. (Figure 3a and 3b). Other cells, including interstitial macrophages (iMphs), dendritic cells (DCs), T cells, B cells, neutrophils and eosinophils, showed comparable numbers between the infected and comorbid groups in both the MavF and AF schemes at 5 w.p.i. (Figure 3). Although infiltration of several immune cell types was slightly attenuated in the comorbid group compared with that in the infected group in the MavF scheme (Supplementary Figure 2a), only lymphocyte and granulocyte infiltration were attenuated in the AF scheme (Supplementary Figure 2b) at 10 w.p.i.

### Attenuation of Allergic Asthma by Prolonged Mav-PI

**Figure 4.**
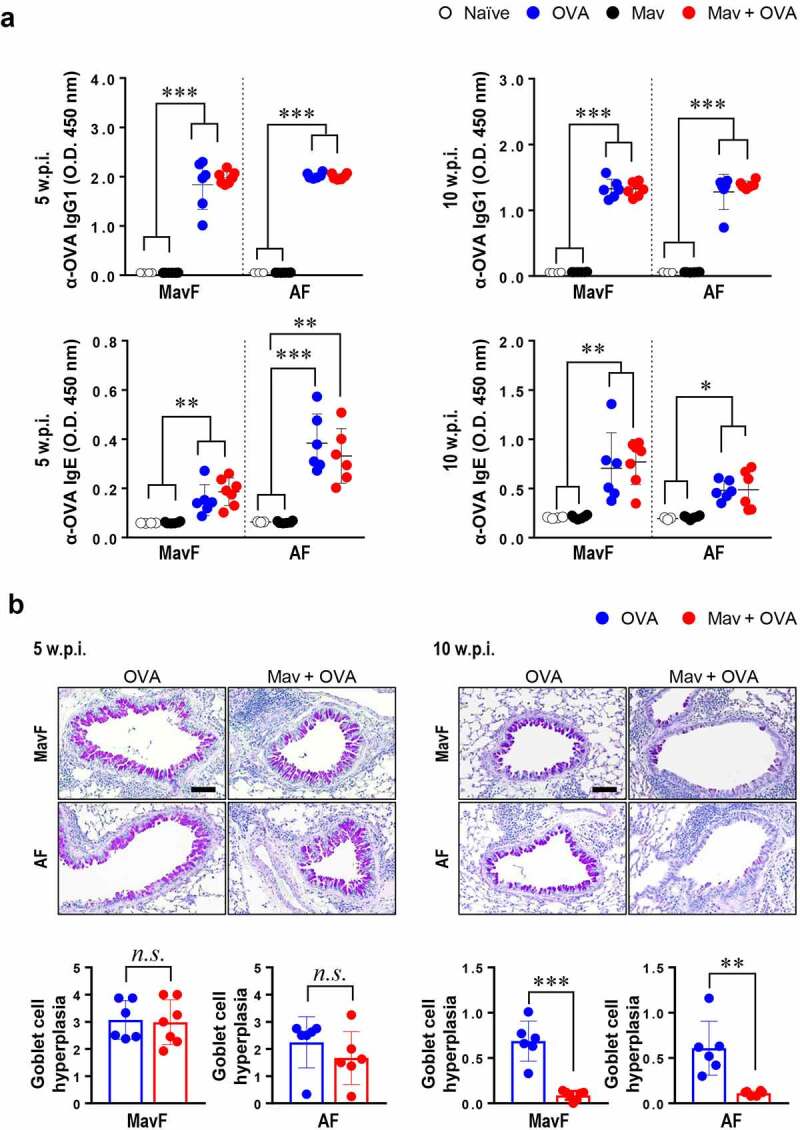
**Phenotypes of ovalbumin (OVA)-induced allergic asthma in BALB/c mice with comorbid *Mycobacterium avium* (Mav) infection**. The OVA-specific humoral responses and the degree of goblet cell hyperplasia in a co-occurring disease model group, in which Mav infection was established before allergic asthma induction (Mav infection first, MavF) or after allergic asthma induction (allergic asthma first, AF), were compared with those in the allergic asthma group. The results of comparisons among the Mav-infected (black), allergic asthma (blue) and comorbid (red) groups are indicated in the figures. (a) OVA-specific IgG1 and IgE in mouse serum were detected. (b) Periodic acid-Schiff (PAS) staining was conducted, and a representative PAS-stained lung section displaying exacerbated goblet cell hyperplasia is shown (original magnification: ×20, scale bar: 100 µm). The levels of goblet cell hyperplasia quantified using ImageJ software are indicated in dot plots under the pictures. Between-group differences were statistically compared by unpaired *t*-tests, and results among more than two groups were compared by one-way analysis of variance followed by Tukey’s multiple comparison test. The statistical significance of the correlation between the number of CFUs and the frequency of T cells was analyzed by linear regression (*p< 0.05, **p< 0.01, ***p< 0.001, *n.s*., not significant)

We then verified that Mav-PI could control the progression of allergic asthma because other mycobacterial species have been reported to alleviate allergic asthma. The humoral reaction to allergen, which was characterized by high levels of OVA-specific IgG1 and IgE, suggested that allergic asthma was well established in both the asthma and comorbid groups at 5 and 10 w.p.i. (Figure 4a). Although the degree of goblet cell hyperplasia was comparable between the allergic asthma and comorbid groups (in both the MavF and AF schemes) at 5 w.p.i., it was markedly reduced in the comorbid group compared with that in the asthma group at 10 w.p.i. (Figure 4b). These results indicate that Mav-PI can alleviate allergic asthma, particularly its histological characteristics. While the bacterial burden and lesion inflammation were exacerbated in the comorbid group, allergic asthma was alleviated by Mav-PI regardless of the preceding disease state.

### Association of the Mav-Specific Th17 Response with the Pathogenesis of Mav-PI in Mice with Comorbid Conditions

Regardless of the sequence of each disease in the comorbid group, we found that Mav infection worsened due to allergic asthma induction. In contrast, allergic asthma was attenuated after Mav infection. Thus, we hypothesized that the host defense system only for controlling Mav-PI would be particularly suppressed under comorbid conditions. To verify this hypothesis, antigen-specific immune responses were investigated by ELISAs and flow cytometry. In the MavF scheme at both 5 and 10 w.p.i. and in the AF scheme at 10 w.p.i., the levels of Mav-specific IL-17A, IFN-γ and TNF-α, important for defense against intracellular pathogens, did not increase under comorbid conditions, in contrast to the result in the infected group (Supplementary Figure 3a and 4). In contrast, in the AF scheme, the IL-17A level in the comorbid group after Mav-PI was higher than that in the infected group at 5 w.p.i. (Supplementary Figure 3b and 4b). Among the analyzed cytokines, only the IL-17A level, which is a critical determinant of intracellular bacterial survival, showed different aspects based on the disease sequence of the comorbid condition. In addition, representative allergic responses, including OVA-specific IL-5 and IL-13 production, were more reduced in the comorbid group than in the allergic asthma group, suggesting that allergic asthma was alleviated in both the MavF and AF schemes at 5 and 10 w.p.i. (Supplementary Figure 3 and 4).

**Figure 5.**
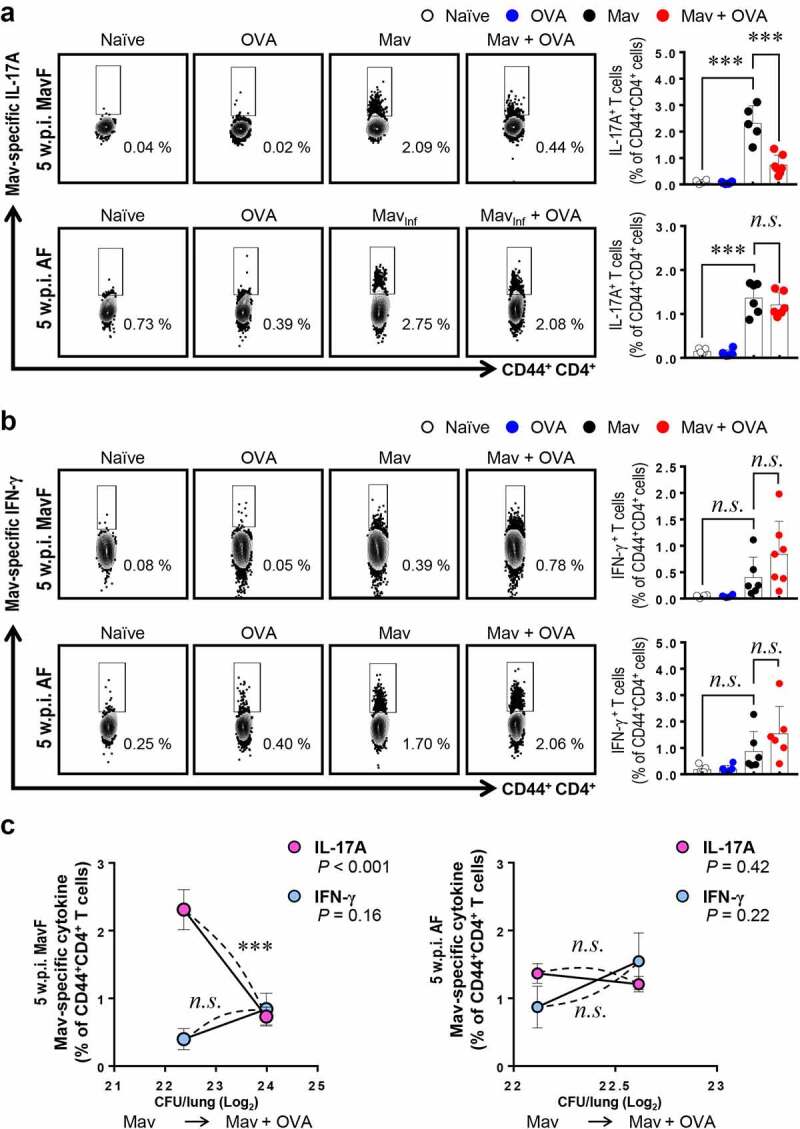
**Attenuation of *Mycobacterium avium* (Mav)-specific cytokine production in CD44^+^CD4^+^ T cells of BALB/c mice in response to stimulation with Mav-specific antigen (Mav_Ag_) at 5 weeks post infection (w.p.i.)**. After stimulation with 10 µg/mL culture filtrate antigen (CFA) from Mav, the functional CD44^+^CD4^+^ T cells in inflamed lungs of the Mav-infected (black) and comorbid (red) groups were evaluated by flow cytometry. (a) The frequencies of IL-17A^+^ cells among CD44^+^CD4^+^ T cells before allergic asthma induction (Mav infection first, MavF) and after allergic asthma induction (allergic asthma first, AF) are indicated in a scatter plot and shown in a bar graph to the right of the gating plot. (b) The frequency of IFN-γ^+^ cells among CD44^+^CD4^+^ T cells in the MavF and AF schemes is indicated in a scatter plot and shown in a bar graph to the right of the gating plot. (c) At 5 w.p.i., a correlation was found according to the preexisting disease under conditions of Mav infection/allergic asthma comorbidity. Mav infection before allergic asthma induction is indicated by MavF (left panel), and allergic asthma induction before Mav infection is indicated by AF (right panel). In each panel, the two left circles represent the Mav-infected group, and the two right circles represent the comorbid group. Between-group differences were statistically compared by unpaired *t*-tests, and results among more than two groups were compared by one-way analysis of variance followed by Tukey’s multiple comparison test. The statistical significance of the correlation between the number of CFUs and the frequency of T cells was analyzed by linear regression (*p< 0.05, **p< 0.01, ***p< 0.001, *n.s*., not significant)

Because T cells are the most well-known initiators of antigen-specific adaptive immunity, we focused on T cell dysfunction after restimulation of cells with Mav_Ag_ (Supplementary Figure 1b). At 5 w.p.i., the frequency of IL-17A^+^CD44^+^CD4^+^ T cells (Th17 cells) was significantly lower in the comorbid group than in the infected group in the MavF scheme but did not differ between these groups in the AF scheme (Figure 5a). Suppressed Th17 cells in the comorbid group in the MavF scheme were maintained up to 10 w.p.i., and those in the comorbid group in the AF scheme were ultimately depleted when the bacterial burden decreased (Supplementary Figure 5a). These results suggest that aggravation of Mav-PI with a suppressed Th17 immune response was more prominent when allergic asthma was induced after infection than vice versa.

On the other hand, the frequency of IFN-γ^+^CD44^+^CD4^+^ T cells (Th1 cells) in the comorbid group was comparable to that in the infected group at 5 w.p.i. and decreased only at 10 w.p.i. (Figure 5b and Supplementary Figure 5b). These results implied that Th17 responses were regulated earlier than Th1 responses in comorbid conditions. To clarify the relationship between T cell responses and bacterial proliferation, we drew a correlation graph between them (Figure 5c and Supplementary Figure 5c). Notably, these results corresponded to the CFU data, which suggested that Th17 cells regulated bacterial growth under conditions of comorbidity, but Th1 cells did not show a significant association with bacterial growth. In summary, modulation of Mav-specific IL-17A production by allergic asthma induction was more crucial for resistance to an increasing bacterial burden than the Mav-specific IFN-γ response.

### Under Conditions of Comorbidity, Severe Mav-PI Disease Phenotypes Were Observed in BALB/c Mice but not C57BL/6 J Mice with a Mav-Specific Th17 Response

**Figure 6.**
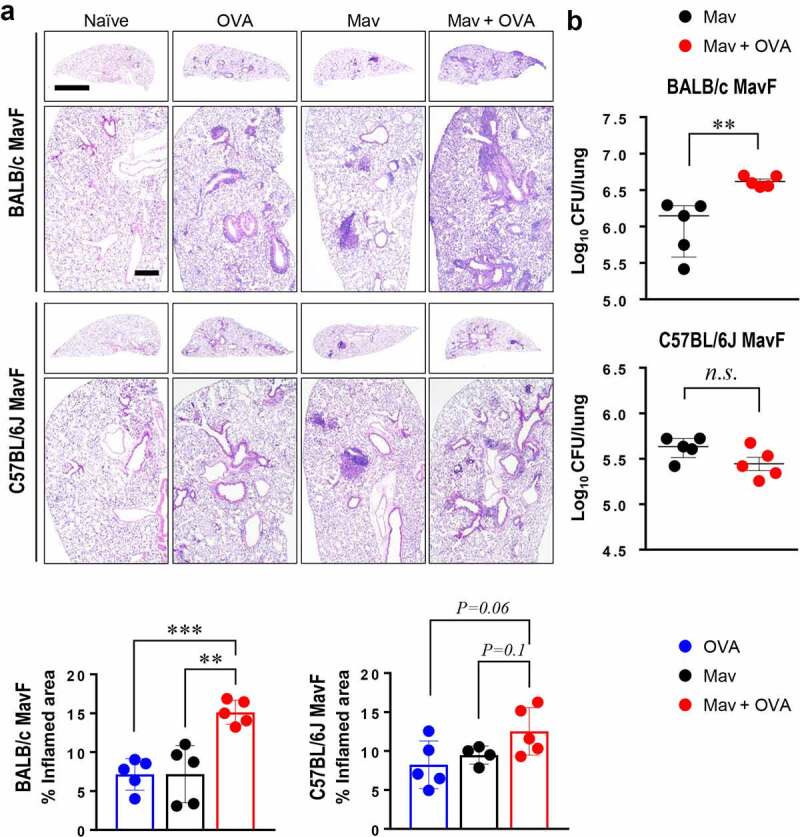
**Comparison of disease phenotypes in the comorbidity schemes between BALB/c and C57BL/6 J mice**. All mice were infected with *Mycobacterium avium* (Mav), as shown in Figure. 1A (Mav infection first, MavF), and sacrificed at 5 weeks post infection (w.p.i.). (a) The right superior lobes of inflamed lungs were stained with hematoxylin and eosin (original magnification: ×1, scale bar: 2 mm; ×4, scale bar: 500 µm), and the inflamed areas of the lungs of the allergic asthma (blue), Mav-infected (black), and comorbid (red) groups were quantified. (b) The number of CFUs in the lungs of each group at 5 w.p.i. are indicated in dot plots. The statistical significance was analyzed by unpaired t-test (*p< 0.05, **p< 0.01, ***p< 0.001, *n.s*., not significant)

**Figure 7.**
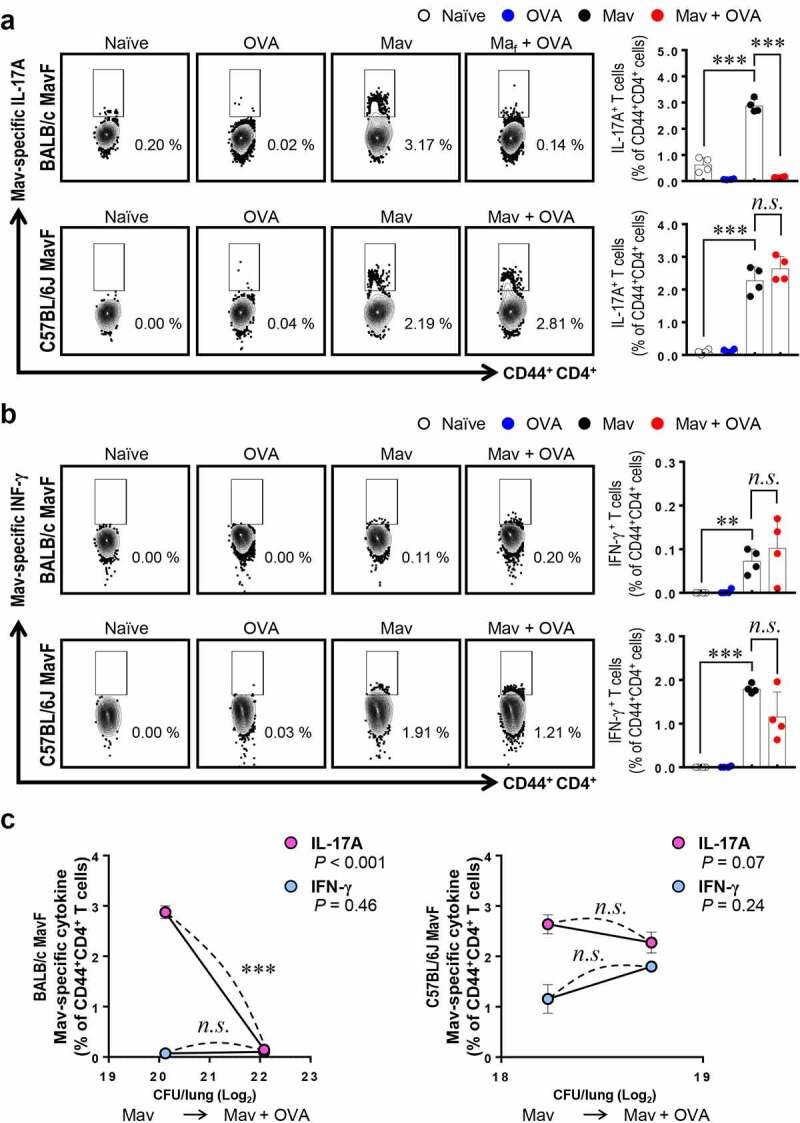
**Comparison of *Mycobacterium avium* (Mav)-specific cytokine production from CD44^+^CD4^+^ T cells in BALB/c mice and C57BL/6 J mice at 5 weeks post infection (w.p.i.)**. After stimulation with 10 μg/mL culture filtrate antigen from Mav (Mav_Ag_), functional CD44^+^CD4^+^ T cells in inflamed lungs, including lungs from mice in the Mav-infected (black) and comorbid (red) groups, were evaluated by flow cytometry. (a) The frequencies of IL-17A^+^ cells among CD44^+^CD4^+^ T cells are indicated in a scatter plot and shown in a bar graph to the right of the gating plot. (b) The frequencies of IFN-γ^+^ cells among CD44^+^CD4^+^ T cells are indicated in a scatter plot and shown in a bar graph to the right of the gating plot. (c) The relationships between the number of CFUs and frequencies of Mav-specific IL-17A and IFN-γ production from CD44^+^CD4^+^ T cells are indicated by a fitted regression line with the corresponding correlation coefficient. In each panel, the two left circles represent the Mav-infected group, and the two right circles represent the comorbid group. The statistical significance was analyzed by unpaired t-test. The statistical significance of the correlation between the number of CFUs and the frequency of T cells was analyzed by linear regression (*p< 0.05, **p< 0.01, ***p< 0.001, *n.s*., not significant)

To determine whether the host’s defense system is activated differently depending on the severity of asthma in the context of comorbidity, we compared two mouse strains, the asthma-susceptible BALB/c strain and the less-susceptible C57BL/6 J strain (Supplementary Figure 6). As shown previously, BALB/c mice showed notably worse lung pathology and faster bacterial growth under conditions of Mav-PI/allergic asthma comorbidity than C57BL/6 J mice, which exhibited comparable phenotypes regardless of the induction of allergic asthma (Figure 6a and 6b). Although differences in immune cell infiltration were observed, the increase in the number of CFUs in each mouse strain was not correlated with immune cell infiltration under conditions of comorbidity (Supplementary Figure 7a and 7b). Interestingly, the frequency of Mav-specific Th17 cells was lower in the comorbid group than in the infected group of BALB/c mice but similar between the comorbid and infected groups of C57BL/6 J mice (Figure 7a). In contrast, the frequencies of Mav-specific Th1 cells were comparable between the comorbid and infected groups for both mouse strains (Figure 7b). The levels of cytokines in the culture supernatants of separated cells from inflamed lungs tended to represent responses similar to those of Mav-specific T cells in both strains (Supplementary Figure. 8).

Overall, the lung inflammation and bacterial growth caused by Mav-PI accompanied by asthma were worse in asthma-susceptible mice (BALB/c) than in less-susceptible mice (C57BL/6 J). In addition, the increase in mycobacterial proliferation in the context of comorbidity was mainly associated with a decrease in Mav-specific Th17 responses at an early stage of infection (Figure 5c and 7c).

## DISCUSSION

The incidence of NTM-PD and allergic asthma has increased worldwide, particularly in middle-aged women with overlapping common clinical symptoms, such as chronic cough, stridor, and wheezing [36]. NTM-PD is both frequently accompanied and aggravated by allergic asthma, and vice versa [12–14,17], but the causes of the observed comorbidity-related exacerbation remain unclear. In this study, the pathological and immunological characteristics of comorbid chronic Mav-PI and allergic asthma were investigated. We discovered conflicting pathogenesis between the two diseases and found that allergic asthma-mediated exacerbation of Mav-PI progression was associated with a significantly reduced Mav-specific Th17 response.

Our study revealed that comorbidities influenced the progression of NTM-PI more than allergic asthma. In particular, the bacterial burden and lung inflammation were markedly aggravated at 5 w.p.i. in the MavF comorbidity scheme (Figure 1b and 1c) and at 10 w.p.i. in the AF comorbidity scheme (Figure 2b and 2c). In contrast, allergic asthma was alleviated in the comorbid group compared with that in the asthma group (Figure 4b). These results suggest that the additional exposure of individuals with preexisting Mav-PI to allergens is responsible for the comorbidity-related exacerbation of Mav-PI pathogenesis. In addition, mycobacterial infections have the potential to regulate allergic asthma by shifting the balance of allergen-specific Th1/Th2 responses [36,37]. An increasing number of studies have demonstrated that Mtb and Bacillus Calmette–Guérin (BCG) exhibit immunotherapeutic potential for asthma [23,38]. However, the effects of allergic asthma on the pathogenesis of mycobacterial infection have received little attention; a few studies have investigated the effects of allergens on Mtb infection but not NTM-PI [25].

Piñeros et al. found that additional exposure of Mtb-infected mice to allergens attenuated Mtb infection, as demonstrated by histopathological analysis, and decreased the number of CFUs at 8 w.p.i., but this finding was not observed in mice with allergic asthma that were later infected with Mtb. These researchers postulated that this finding was due to an unexpected bactericidal function of M2-type macrophages in proinflammatory conditions [25], but they did not investigate T cell responses. In the present study, we obtained the opposite results, and we focused on T cells rather than infiltrated myeloid cell populations because these cells showed comparable lung infiltration between the Mav-PI and comorbid groups (Figure 3 and Supplementary Figure 1 and 2). In addition, a study by H. Tomioka et al. revealed that activated T cell-mediated activation of suppressive macrophages is critical for Mav control [39]. We therefore focused on the Mav growth-inhibiting function of T cells under conditions of comorbidity. Strikingly, the increase in the number of CFUs in the comorbid group was correlated with a significant reduction in Th17 cell levels but not in Th1 cell levels at 5 w.p.i. in the MavF scheme and at 10 w.p.i. in the AF scheme (Figure 5 and Supplementary Figure 5), which corresponded to the most exacerbated phenotypes (Figure 1 and 2). The levels of Mav-specific IL-17A production were also inversely correlated with the bacterial burden (Supplementary Figure 3 and 4). To clarify the mechanism by which allergic asthma exacerbated Mav-PI pathogenesis, we compared BALB/c and C57BL/6 J mice, which typically show different susceptibilities to allergic asthma (Figure 6 and Supplementary Figure 6) [40]. Consistent with the abovementioned results, the increase in the number of CFUs was more correlated with Th17 cells than with Th1 cells, but this correlation was observed only in BALB/c mice (Figure 7c).

Similar to our results, infection with intracellular pathogens of the *Brucella* family is reportedly exacerbated by exposure to allergens via IL-4/STAT6 signaling. However, infection with *Streptococcus pneumoniae*, an extracellular pathogen, is alleviated by allergen exposure, whereas Mtb infection is not altered under conditions of comorbidity [26]. Monin et al studied the effect of *Aspergillus fumigatus*, which commonly coinfects NTM, on *Mycobacterium abscessus* infection. Comorbidity resulting from preexisting infection with *A. fumigatus* followed by infection with *M. abscessus* resulted in upregulated *M. abscessus*-specific Th17 responses, resulting in better control of *M. abscessus* infection, and this effect was supported by an elevated bacterial burden observed in IL-17ra^−/-^ mice [41]. Similarly, in the AF scheme of the current study, lung IL-17A levels were increased in the comorbid group at 5 w.p.i. (Supplementary Figure 3b), which resulted in failure of Mav growth control (Figure 1c). Collectively, our current findings suggest that the exacerbation of Mav-PI in the comorbid group might have occurred because Th2 immune responses followed by allergen exposure can modulate the Th17 response, particularly under conditions of comorbidity. In fact, most exacerbations of asthma induced by viral and bacterial infections are thought to accompany Th2 and Th17 responses [42,43]. However, a recent clinical study demonstrated that Th2 immunity negatively regulates the Th17 response in asthmatic patients and that experimental anti-Th2 interventions markedly upregulate IL-17 gene expression while enriching IL-17^+^CD4^+^ T cell populations in the lungs and draining lymph nodes in a preclinical model of chronic asthma [44]. Thus, the increased IL-17A expression in the comorbid group compared with that in the asthma group in the present study (Supplementary Figure 3 and 4) might have been responsible for the attenuation of allergic asthma in the comorbid group. Therefore, further studies on the precise mechanisms through which Th2 and Th17 responses counter-regulate one another in the lungs under conditions of NTM-PI/asthma comorbidity are needed to support clinical applications.

It is especially notable that the increasing bacterial burden due to attenuated Th17 cells found in the present study was not correlated with lung pathology (e.g., the pathology in the AF scheme at 10 w.p.i. did not show exacerbated lesions). This result is in line with previous studies that showed that immunodeficient mice or IFN-γ^−/-^ mice developed increased bacterial burdens without exacerbated lung pathology and that failure of granuloma formation via macrophages and lymphocytes made the mice susceptible to Mav infection [45–47]. Therefore, we postulate that our results at 10 w.p.i. were due to attenuated Th17 and Th1 cell responses causing failure of inflammatory cells to infiltrate the lung, thereby leading to less aggravation of lung inflammation in the comorbid group than in the infected group in the AF scheme at 10 w.p.i [48].

To the best of our knowledge, this study provides the first demonstration that Mav-specific Th17 responses are crucial for controlling NTM-PI comorbid with allergic asthma, although whether both Th1 and Th17 responses are important for controlling Mav-PI is controversial [49–51]. This finding is in agreement with previous reports indicating that IL-17 levels are suppressed in patients with NTM-PD [49] and that Th17 responses are important for controlling the progression of mycobacterial infection [34]. We thus propose several mechanisms for the allergic asthma-induced attenuation of Mav-specific Th17 cell responses. First, antigen diversity confounds the recognition and interception of Mav-specific defense responses [52,53]. Second, increases in regulatory T cell numbers to control hyperinflammation in the context of comorbidity can decrease Th17 cell numbers to preserve host homeostasis [54]. Third, several studies have clarified that allergen-induced Th2-type cytokines can suppress Th17 responses [44,55], which supports our finding that only relatively late induction of allergic asthma inhibited preexisting Mav-specific Th17 responses and increased the number of CFUs at 5 w.p.i.

Further investigations are needed to clarify these possibilities. First, whether other allergic asthma-related factors regulate Mav-specific responses should be investigated. For example, Th2 cytokines, such as IL-33, that are secreted upon OVA exposure can promote T cell activity [56]. Third, although we analyzed immune cell populations with a particular focus on antigen-specific T cell responses in the present study, further investigation is needed to define the mechanism by which Th17 immune responses were inhibited under conditions of comorbidity. This mechanism might involve the functions of DCs, which can regulate T cell responses. Furthermore, Mav-specific Th17 responses should be confirmed with inhibition studies. Moreover, we have yet to examine other features of asthma, such as airway hyperresponsiveness.

Despite its limitations, the present study significantly enhances the understanding of Mav-PI pathogenesis based on relevant alterations in disease-specific immune responses, particularly in the context of comorbidity with allergic asthma. To the best of our knowledge, this study provides the first demonstration that Mav-specific Th17 responses are disrupted by allergic asthma, resulting in exacerbated Mav-PI. These results suggest that Mav-specific Th17 responses are important for controlling pathogenesis under conditions of comorbidity. We expect that this study will contribute to further identification of effective therapeutics for NTM-PI, particularly in the presence of other Th2-related diseases.

## Supplementary Material

Supplemental Material

## Data Availability

The datasets used and/or analyzed during the current study are available from the corresponding author on reasonable request.
