# Supplementary material for: Exacerbation of Mycobacterium avium pulmonary infection by comorbid allergic asthma is associated with diminished mycobacterium-specific Th17 responses
Source: Virulence. 2021 Oct 4;12(1):2546–61. doi: 10.1080/21505594.2021.1979812 (PMC8496529; doi:10.1080/21505594.2021.1979812)
Supplement: Supplemental Material [file KVIR_A_1979812_SM9631.zip › supplementary/Revised_Supple-Data_KVIR-2021-0115clean.docx]

**Exacerbation of *Mycobacterium avium* Pulmonary Infection by Comorbid Allergic Asthma Is Associated with Diminished Mycobacterium-Specific Th17 Responses**

**Running title:** Mav-PI exacerbation by allergic asthma

Yeeun Bak^1,2^**^†^**, Sang Chul Park^3^**^†^**, Dahee Shim^1^, Yura Ha^1,2^, Jumi Lee^1,2^, Hongmin Kim^1,2^, Kee Woong Kwon^1^, Joo-Heon Yoon^4,5,6*^, and Sung Jae Shin^2,5,7*^

^1^Department of Microbiology, Yonsei University College of Medicine, Seoul, Korea

^2^Brain Korea 21 Program for Leading Universities and Students (PLUS) Project for Medical Science, Yonsei University College of Medicine, Seoul, Korea

^3^Department of Otorhinolaryngology-Head and Neck Surgery, Kangnam Sacred Heart Hospital, Hallym University College of Medicine, Seoul, Korea

^4^Department of Otorhinolaryngology, Yonsei University College of Medicine, Seoul, Korea

^5^Global Research Laboratory for Allergic Airway Diseases, Yonsei University College of Medicine, Seoul, Korea

^6^The Airway Mucus Institute, Yonsei University College of Medicine, Seoul, Korea

^7^Department of Microbiology, Institute for Immunology and Immunological Diseases, Yonsei University College of Medicine, Seoul, Korea

**†**These authors contributed equally to this work.

**Online Supplementary Data**

This Online Supplementary Data file includes:

Supplementary Methods

Supplementary Tables

Supplementary Reference

Supplementary Figure Legends

**Supplementary Methods**

**Preparation of Culture Filtrate Antigen from *Mycobacterium avium* (Mav_Ag_)**

To obtain Mag_Ag_, concentrated bacteria were obtained at the mid-logarithmic phase after 8 weeks of incubation in protein-free modified Watson-Reid medium (mWR medium, pH 6.0) at 37°C. After the medium was removed by centrifugation at 30000 × *g* for 30 min, the sediments were filtered through a 0.2-µm-pore-size filter (Nalge Nunc International). The concentrate was obtained using a Centricon Plus (5-kDa molecular weight cutoff; Amicon, Sigma-Aldrich) and dialyzed five times in 10 mM phosphate-buffered saline (PBS, pH 7.2) using a Slide-A-Lyzer Dialysis Cassette (Pierce). The antigen concentration was determined using a protein assay kit (Bio-Rad Laboratories) [1].

**Animal Experiment Design**

Four mouse groups, including the naïve, ovalbumin (OVA)-induced allergic asthma, *Mycobacterium avium* (Mav)-infected, and comorbid (Mav + OVA) groups, were investigated. The mice in the infected group were aerogenically infected with predetermined numbers of Mav using a Glas-Col chamber (Terre Haute). Allergic asthma and Mav infection were induced in the comorbid group using two independent experimental designs. Based on the time point of Mav infection, the mice in the comorbid groups were separated into Mav first (MavF, Mav infection before allergic asthma induction) and asthma first (AF, Mav infection after allergic asthma induction) schemes. The mice in the MavF scheme were first infected with Mav and then subjected to allergic asthma induction to establish comorbidities. The mice were sensitized twice via intraperitoneal (i.p.) injection of 50 µg of OVA (grade V; Sigma-Aldrich) and 1.32 mg of aluminum hydroxide (Alum, Thermo Scientific) in 200 μL of 1× Dulbecco’s PBS (dPBS, Biowest). After one week, the mice were administered an airway allergen challenge via exposure to OVA aerosols for 20 min three times per week for 2 weeks. In the AF scheme, allergic asthma was first induced as previously described, and the mice were then infected with Mav. All mice were sacrificed at 5 and 10 weeks post infection (w.p.i.) (Figs. 1A and 2A).

**Intracellular Cytokine Staining for Classification of T Cell Subsets by Flow Cytometry**

To analyze cytokine-specific T cell subsets, suspended single cells at a density of 2.5 × 10^6^ cells/mL were restimulated with 10 µg/mL OVA or 10 µg/mL Mav_Ag_ with 2 µg/mL BD Golgi-Plug (BD) and incubated for 12 h at 37°C in 5% CO_2_. After the surface molecules of single cells from the lungs were stained (Supplementary Table 1), the cells were fixed and permeabilized for 40 min at 4°C, and intracellular targets were then stained. The stained cells were then washed, fixed with fixation buffer (Invitrogen) and analyzed by flow cytometry using FlowJo software (TreeStar, Inc.). The details of the antibodies used are provided in Supplementary Table 1, and representative flow cytometry plots are shown in Supplementary Fig. 1B.

**Supplementary Table 1.** Antibodies used for flow cytometry analysis

| **Target** | **Clone** | **Fluorochrome** | **Vender** | **Cat. #** |
| --- | --- | --- | --- | --- |
| Dead cells | - | Far Red | Invitrogen | L23102 |
| CD90.2 | 53-2.1 | Brilliant Violet 605 | BioLegend | 140318 |
| CD8α | 53-6.7 | Brilliant Violet 785 | BioLegend | 100750 |
| CD4 | RM4-5 | PerCP-Cy5.5 | BD | 550954 |
| CD44 | IM7 | V450 | BD | 560451 |
| IL-17A | TC11-18H10 | Alexa Fluor 488 | BD | 560220 |
| IFN-γ | XMG1.2 | PE | BD | 554412 |
| CD45 | 30-F11 | BV421 | BD | 563890 |
| F4/80 | BM8 | PE-Cyanine7 | Invitrogen | 25-4801-82 |
| Siglec-F | E50-2440 | APC-R700 | BD | 565183 |
| CD64 (FcγRI) | X54-5/7.1 | PE | BioLegend | 139304 |
| CD11b | M1/70 | PerCP-Cyanine5.5 | Invitrogen | 45-0112-82 |
| CD11c | N418 | PE/Dazzle 594 | BioLegend | 117348 |
| I-A/I-E (MHC class II) | M5/114.15.2 | APC/Cyanine7 | BioLegend | 107628 |
| Ly-6G | 1A8 | BV711 | BD | 563979 |
|  | 1A8 | Brilliant Violet 785 | BioLegend | 127645 |
| CD19 | eBio1D3 (1D3) | FITC | Invitrogen | 11-0193-82 |

**Supplementary Table 2.** Antibodies used for ELISAs

| **Target** | **Gene ID** | **Sensitivity** | **Vender** | **Cat. #** |
| --- | --- | --- | --- | --- |
| IL-17A (homodimer) | 16171 | 4 pg/mL | Invitrogen | 88-7371-88 |
| IFN-γ | 15978 | 15 pg/mL | Invitrogen | 88-7314-88 |
| TNF-α | 21926 | 8 pg/mL | Invitrogen | 88-7324-88 |
| IL-5 | 16191 | 4 pg/mL | Invitrogen | 88-7054-88 |
| IL-13 | 16163 | 4 pg/mL | Invitrogen | 88-7137-88 |
| IL-10 | 16153 | 16 pg/mL | BioLegend | 431414 |

**Supplementary Reference**

[1] Shin AR, Kim HJ, Cho SN, et al. Identification of seroreactive proteins in the culture filtrate antigen of *Mycobacterium avium* ssp. *paratuberculosis* human isolates to sera from Crohn's disease patients. FEMS Immunol Med Microbiol 2010;58:128-137.

**Supplementary Figure Legends**

**Supplementary Figure 1. Flow cytometry gating strategy for the identification of various immune cell populations and functional T cells.** (A) Single live cells in a population of lung cells were manually gated to identify neutrophils, T cells, eosinophils, alveolar macrophages (aMphs), interstitial macrophages (iMphs), dendritic cells (DCs) and B cells. (B) To analyze the cytokines secreted by CD90.2^+^ T cells, isolated single live lung cells were restimulated with *Mycobacterium avium* (Mav)-derived antigen. IFN-γ and IL-17A secreted from CD44^+^CD4^+^ T cells are indicated in the main figure.

**Supplementary Figure 2. Alterations in the cellular composition of inflamed lungs due to *Mycobacterium avium* (Mav) infection co-occurring with allergic asthma at 10 weeks post infection (w.p.i.).** Myeloid and lymphoid cell populations in the lungs were assessed by flow cytometry. The counts of alveolar macrophages (aMphs), interstitial macrophages (iMphs), dendritic cells (DCs), B cells, T cells, neutrophils, and eosinophils were compared between the Mav-infected and comorbid groups. (A) Mice that were infected with Mav before allergic asthma induction (red, Mav infection first, MavF) were sacrificed at 10 w.p.i. and compared with mice belonging to the naïve (white), allergic asthma (blue) and Mav-infected (black) groups. (B) The infiltrated immune cells in the inflamed lungs of mice infected with Mav after allergic asthma induction (allergic asthma first, AF) were analyzed by flow cytometry. The compositions of inflammatory lung cells in the infected and comorbid groups are displayed in two dimensions based on t-stochastic neighbor embedding (t-SNE) to facilitate comparison. Between-group differences were statistically compared by unpaired *t*-tests, and results among more than two groups were compared by one-way analysis of variance followed by Tukey’s multiple comparison test (**p*<0.05, ***p*<0.01, ****p*<0.001, *n.s.*, not significant).

**Supplementary Figure 3. Comparison of cytokine secretion from inflamed lung cells reflecting antigen-specific responses at 5 weeks post infection (w.p.i.).** Separated lung cells from the conditioned mice were cultured for 12 h with 10 µg/mL *Mycobacterium avium* (Mav)-derived antigen (Mav_Ag_) or 10 µg/mL ovalbumin (OVA_Ag_). (A) Allergic asthma was induced in mice that were previously infected with Mav (Mav infection first, MavF). (B) Allergic asthma was induced in mice before Mav infection (allergic asthma first, AF). Antigen-specific cytokine production was analyzed by ELISAs. Each comorbid group (red) was analyzed and compared with the corresponding control groups, namely, a naïve control group (white) and single-disease control groups (allergic asthma, blue; Mav infection, black). The comorbid group was statistically compared with the corresponding single-disease groups by unpaired t-tests (**p*<0.05, ***p*<0.01, ****p*<0.001, *n.s.*, not significant, *n.d*., not detected).

**Supplementary Figure 4. Comparison of cytokine secretion from inflamed lung cells reflecting antigen-specific responses at 10 weeks post infection (w.p.i.).** Separated lung cells from the conditioned mice were cultured for 12 h with 10 µg/mL *Mycobacterium avium* (Mav)-derived antigen (Mav_Ag_) or 10 µg/mL ovalbumin (OVA_Ag_). (A) Allergic asthma was induced in mice that were previously infected with Mav (Mav infection first, MavF). (B) Allergic asthma was induced in mice before Mav infection (allergic asthma first, AF). Antigen-specific cytokine production was analyzed by ELISAs. Each comorbid group (red) was analyzed and compared with the corresponding control groups, namely, a naïve control group (white) and single-disease control groups (allergic asthma, blue; Mav infection, black). The comorbid group was statistically compared with the corresponding single-disease groups by unpaired t-tests (**p*<0.05, ***p*<0.01, ****p*<0.001, *n.s.*, not significant, *n.d*., not detected).

**Supplementary Figure 5. Attenuation of *Mycobacterium avium* (Mav)-specific cytokine production in CD44^+^CD4^+^ T cells in the comorbid group compared with that in the infected group at 10 weeks post infection (w.p.i.)*.*** After stimulation of separated lung cells with 10 µg/mL Mav-derived antigen, CD44^+^CD4^+^ T cells in the inflamed lungs of the mice from the naïve (white), allergic asthma (blue), Mav-infected (black) and comorbid (red) groups were evaluated by flow cytometry. (A) The frequency of IL-17A^+^ T cells among CD44^+^CD4^+^ T cells is indicated in the scatter bar graphs. (B) The frequency of IFN-γ^+^ T cells among CD44^+^CD4^+^ T cells is presented as a scatter plot with a dot plot. (C) The Mav infection before allergic asthma induction (Mav infection first, MavF) scheme (left panel) and allergic asthma induction before Mav infection (allergic asthma first, AF) scheme (right panel) were compared after continuous challenge with allergen at 10 w.p.i. In each panel, the two left circles represent the Mav-infected group, and the two right circles represent the comorbid group. Between-group differences were statistically compared by unpaired *t*-tests, and results among more than two groups were compared by one-way analysis of variance followed by Tukey’s multiple comparison test. The statistical significance of the correlation between the number of CFUs and the frequency of T cells was analyzed by linear regression (**p*<0.05, ***p*<0.01, ****p*<0.001, *n.s.*, not significant).

**Supplementary Figure 6. Characteristics of ovalbumin (OVA)-induced allergic asthma in *Mycobacterium avium* (Mav)-infected BALB/c and C57BL/6J mice.** (A) Periodic acid-Schiff (PAS) staining was performed, and a representative PAS-stained lung section showing exacerbation of goblet cell hyperplasia is shown (original magnification: ×20, scale bar: 100 µm). Comparisons of the allergic asthma (blue) and comorbid (red) groups are indicated in the scatter bar graphs. (B) OVA-specific IgG1 and IgE in mouse serum were detected. Between-group differences were statistically compared by unpaired *t*-tests, and results among more than two groups were compared by one-way analysis of variance followed by Tukey’s multiple comparison test (**p*<0.05, ***p*<0.01, ****p*<0.001, *n.s.*, not significant).

**Supplementary Figure 7. Cellular compositions in individual BALB/c and C57BL/6J mice at 5 weeks post infection (w.p.i.).** Representative alterations in the cell compositions of different mouse strains with different susceptibilities to allergic asthma are shown. Alveolar macrophages (aMphs), interstitial macrophages (iMphs), dendritic cells (DCs), B cells, T cells, neutrophils and eosinophils in each group were analyzed by flow cytometry. (A) BALB/c mice, which are vulnerable to allergic asthma, were challenged experimentally. (B) C57BL/6J mice, in which allergic asthma cannot be easily induced, were also challenged experimentally. To verify the difference between the *Mycobacterium avium* (Mav)-infected and comorbid groups, the t-stochastic neighbor embedding (t-SNE) results for each cell population were merged between these two groups. The allergic asthma group (blue) was compared with the naïve group (white), and the comorbid group (red) was compared with the Mav-infected group (black). The comorbid group was established using the MavF scheme (Mav infection before allergic asthma induction), as indicated in Fig. 1. Between-group differences were statistically compared by unpaired *t*-tests, and results among more than two groups were compared by one-way analysis of variance followed by Tukey’s multiple comparison test (**p*<0.05, ***p*<0.01, ****p*<0.001, *n.s.*, not significant).

**Supplementary Figure 8.** **Comparison of cytokine secretion from inflammatory lung cells in response to a single disease-specific antigen, *Mycobacterium avium* (Mav)-derived antigen (Mav_Ag_) or ovalbumin (OVA_Ag_), according to the mouse strain at 5 weeks post infection (w.p.i.).** Separated lung cells from conditioned mice were stimulated with 10 µg/mL Mav_Ag_ or 10 µg/mL OVA_Ag_ for 12 h. Representative cytokines in the supernatant of lung cells incubated under inflammatory conditions were analyzed by ELISAs. Under conditions of comorbidity, allergic asthma was induced in the mice. (A) BALB/c mice, which are susceptible to allergic asthma, were used as a representative model and challenged under the conditions described below. (B) In contrast to BALB/c mice, C57BL/6J mice were used as a mouse model that is resistant to allergic asthma. Each comorbid group (red) was analyzed and compared with the corresponding control groups, namely, the naïve group (white) and single-disease control groups (asthma, blue; Mav infection, black). The comorbid group was statistically compared with the corresponding single-disease groups by unpaired t-tests (**p*<0.05, ***p*<0.01, ****p*<0.001, *n.s.*, not significant, *n.d*., not detected).
