# Supplementary figures and images for: Exacerbation of Mycobacterium avium pulmonary infection by comorbid allergic asthma is associated with diminished mycobacterium-specific Th17 responses
Source: Virulence. 2021 Oct 4;12(1):2546–61. doi: 10.1080/21505594.2021.1979812 (PMC8496529; doi:10.1080/21505594.2021.1979812)

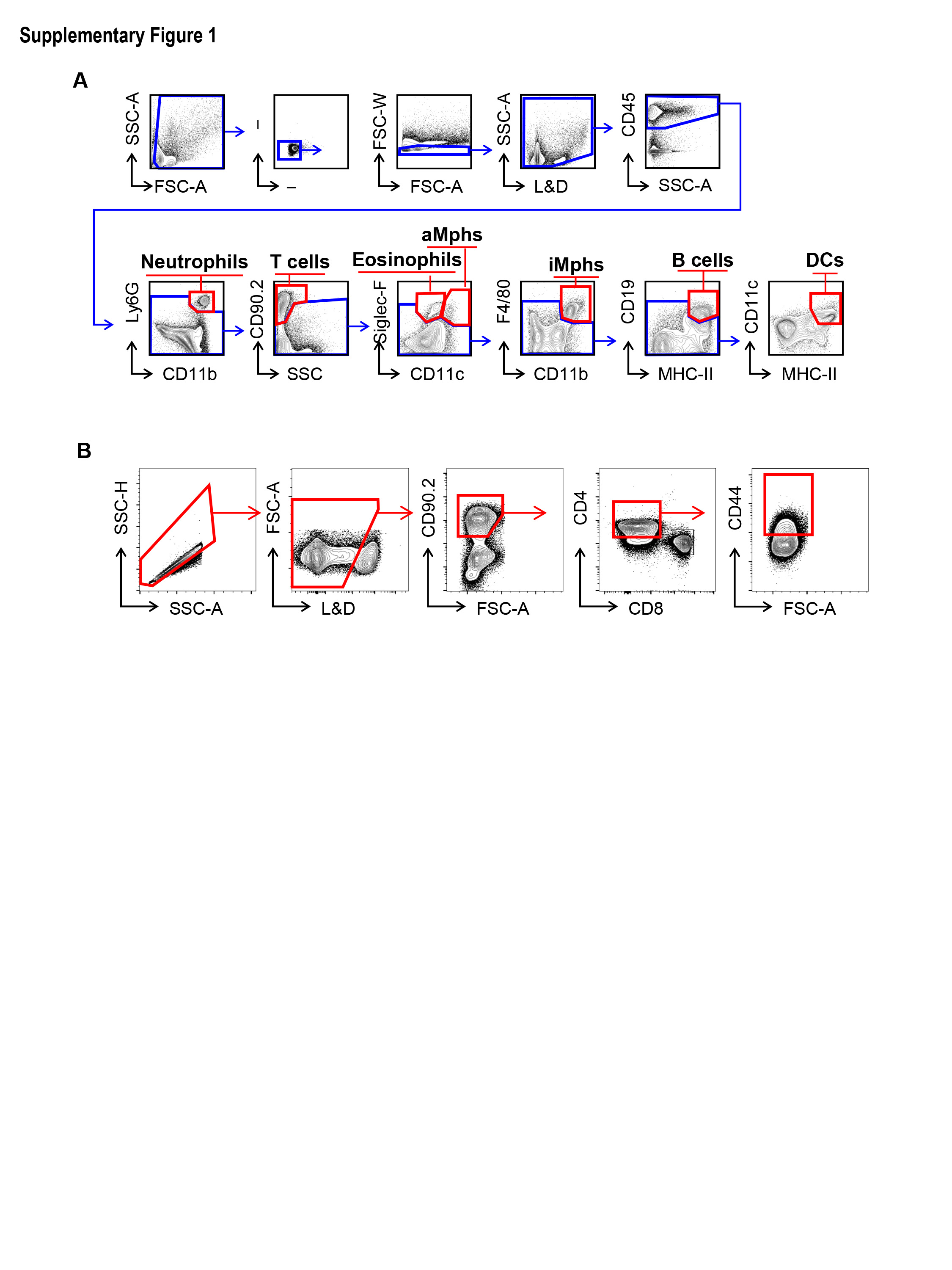

Supplement: Supplemental Material [file KVIR_A_1979812_SM9631.zip › supplementary/Revised_Supple-Figure1_KVIR-2021-0115.jpg]

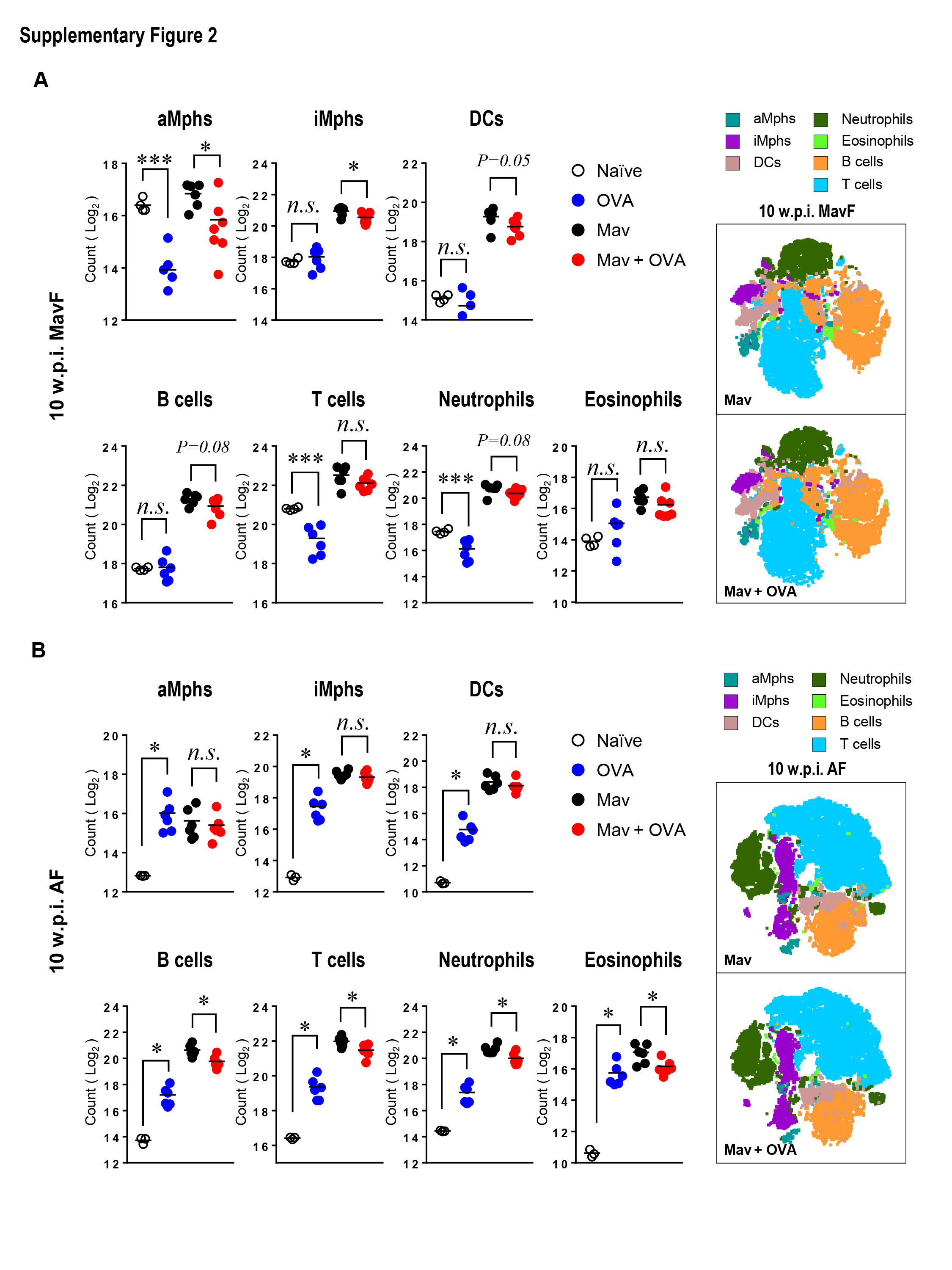

Supplement: Supplemental Material [file KVIR_A_1979812_SM9631.zip › supplementary/Revised_Supple-Figure2_KVIR-2021-0115.jpg]

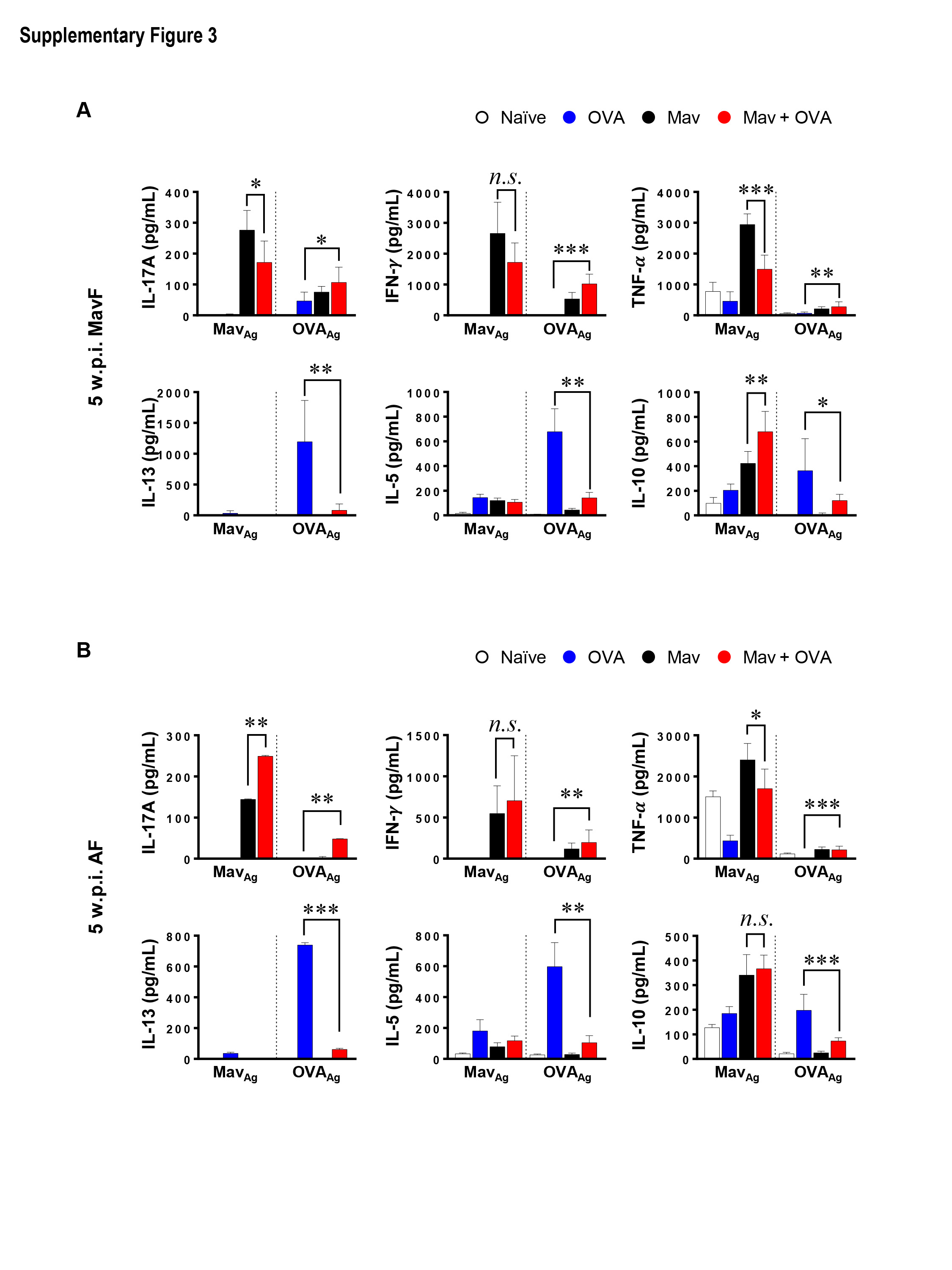

Supplement: Supplemental Material [file KVIR_A_1979812_SM9631.zip › supplementary/Revised_Supple-Figure3_KVIR-2021-0115.jpg]

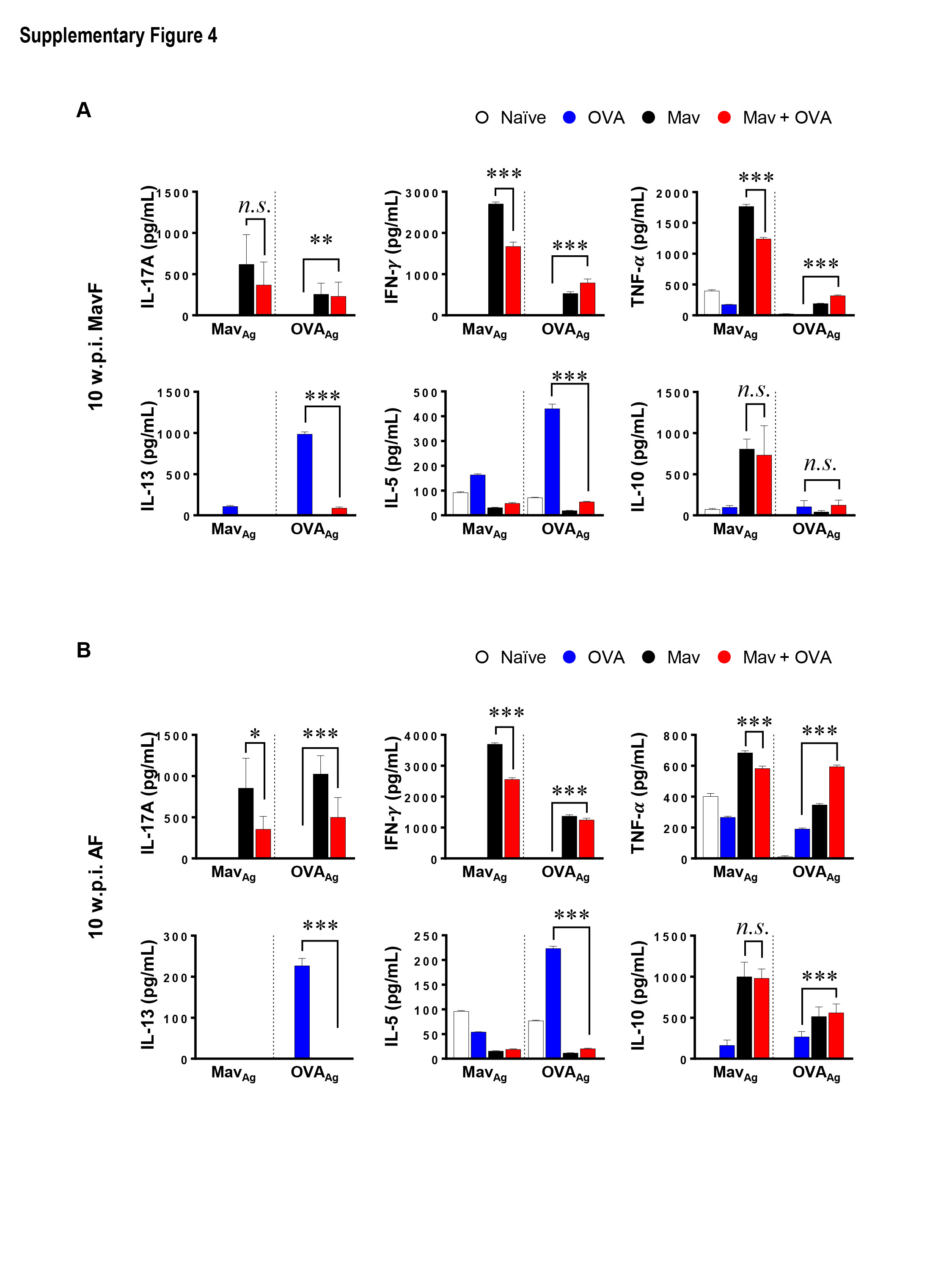

Supplement: Supplemental Material [file KVIR_A_1979812_SM9631.zip › supplementary/Revised_Supple-Figure4_KVIR-2021-0115.jpg]

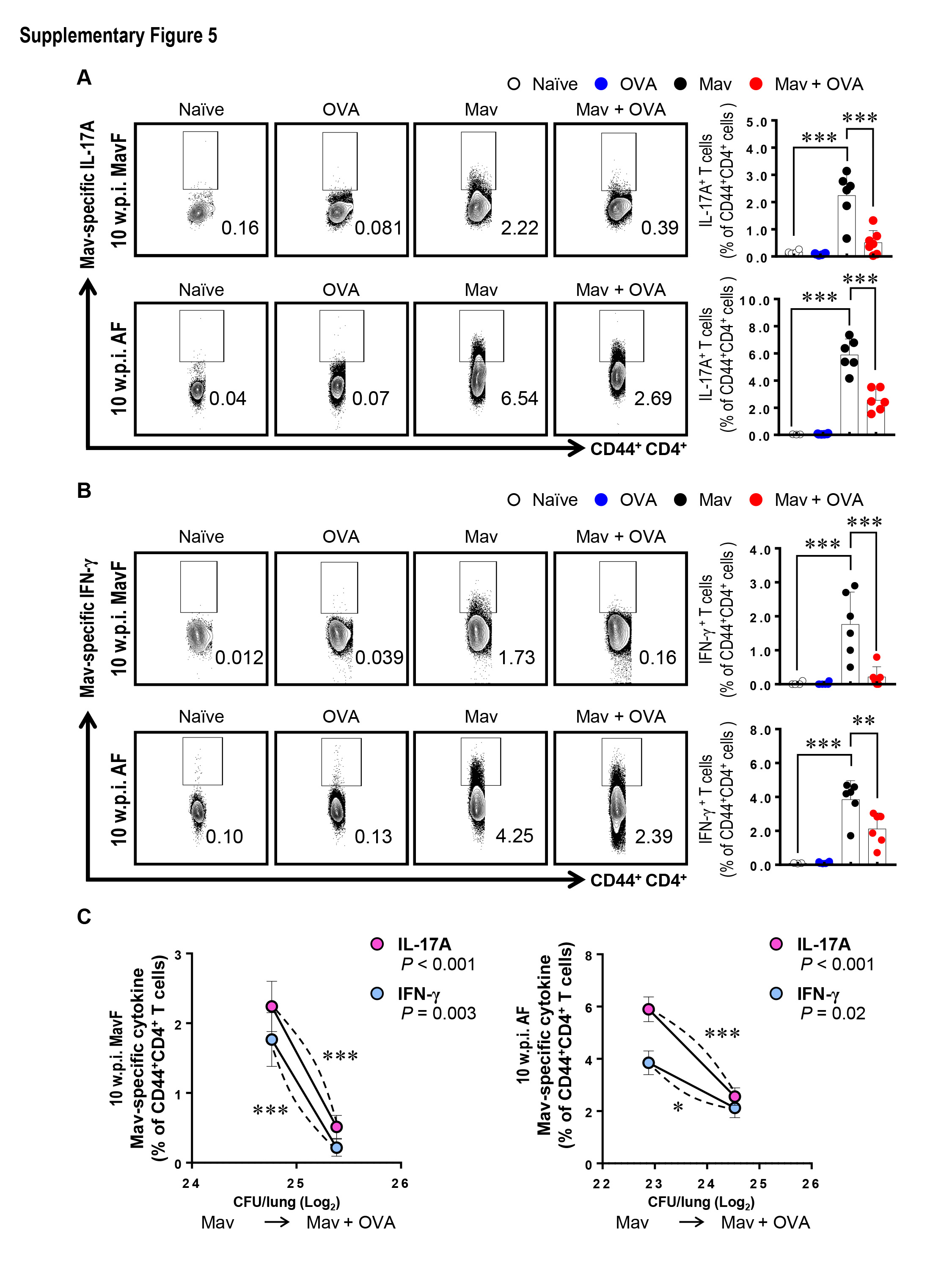

Supplement: Supplemental Material [file KVIR_A_1979812_SM9631.zip › supplementary/Revised_Supple-Figure5_KVIR-2021-0115.jpg]

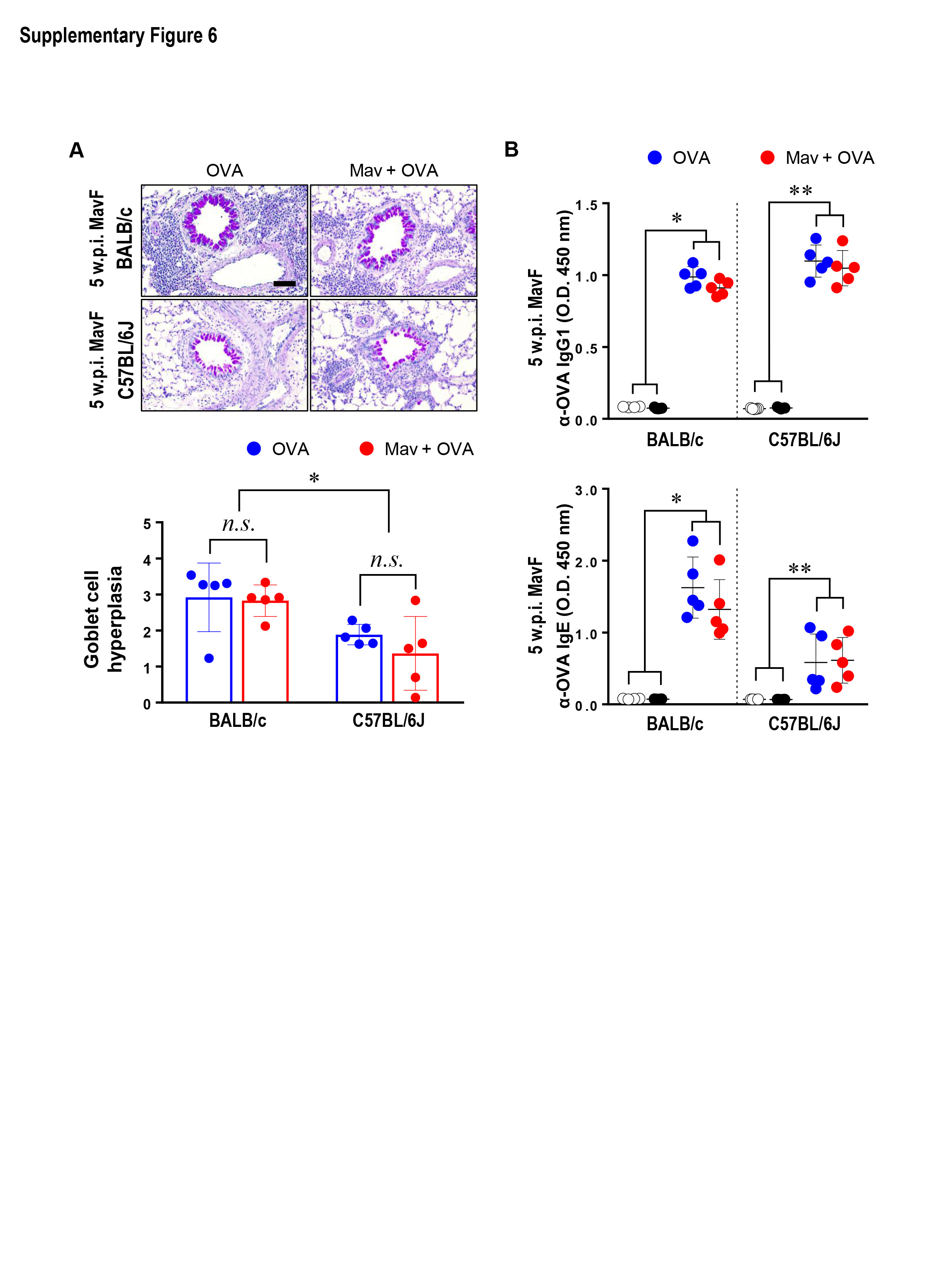

Supplement: Supplemental Material [file KVIR_A_1979812_SM9631.zip › supplementary/Revised_Supple-Figure6_KVIR-2021-0115.jpg]

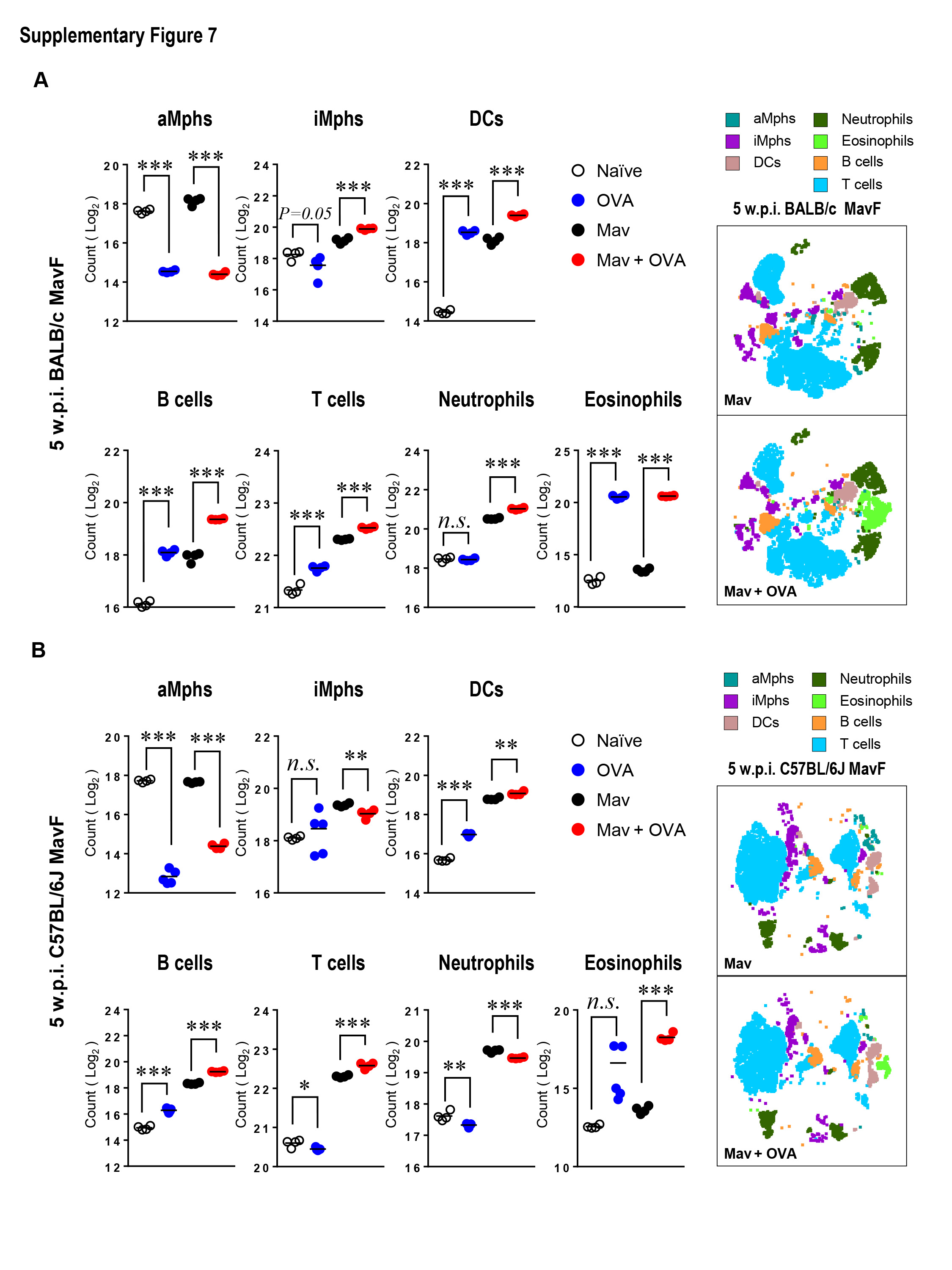

Supplement: Supplemental Material [file KVIR_A_1979812_SM9631.zip › supplementary/Revised_Supple-Figure7_KVIR-2021-0115.jpg]

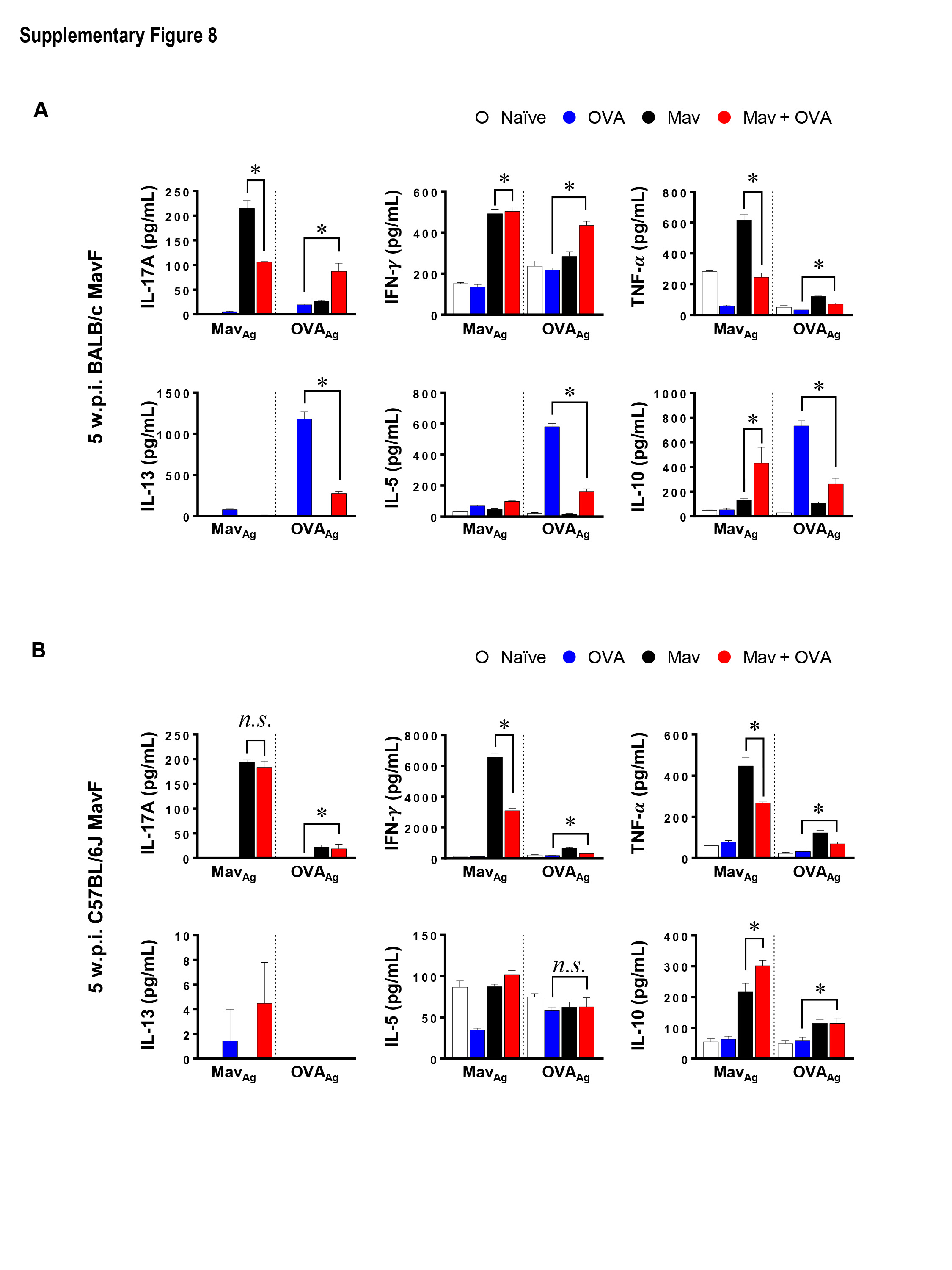

Supplement: Supplemental Material [file KVIR_A_1979812_SM9631.zip › supplementary/Revised_Supple-Figure8_KVIR-2021-0115.jpg]
